# Artificial intelligence in knee arthroplasty: current concept of the available clinical applications

**DOI:** 10.1186/s42836-022-00119-6

**Published:** 2022-05-02

**Authors:** Cécile Batailler, Jobe Shatrov, Elliot Sappey-Marinier, Elvire Servien, Sébastien Parratte, Sébastien Lustig

**Affiliations:** 1 Orthopaedic Surgery and Sports Medicine Department, Croix-Rousse Hospital, Lyon University Hospital, Lyon, France; 2 Univ Lyon, Claude Bernard Lyon 1 University, IFSTTAR, LBMC UMR_T9406, F69622 Lyon, France; 3 Sydney Orthopedic Research Institute, University of Notre Dame Australia, Hornsby and Ku-Ring Hospital, Sydney, Australia; 4 LIBM – EA 7424, Interuniversity Laboratory of Biology of Mobility, Claude Bernard Lyon 1 University, Lyon, France; 5 International Knee and Joint Centre, Abu Dhabi, United Arab Emirates; 6 Institute for Locomotion, Aix-Marseille University, Marseille, France

**Keywords:** Knee arthroplasty, Artificial intelligence, Machine learning, Predictive models, Augmented reality, Robotic surgery

## Abstract

**Background:**

Artificial intelligence (AI) is defined as the study of algorithms that allow machines to reason and perform cognitive functions such as problem-solving, objects, images, word recognition, and decision-making. This study aimed to review the published articles and the comprehensive clinical relevance of AI-based tools used before, during, and after knee arthroplasty.

**Methods:**

The search was conducted through PubMed, EMBASE, and MEDLINE databases from 2000 to 2021 using the 2009 Preferred Reporting Items for Systematic Reviews and Meta-Analyses Protocol (PRISMA).

**Results:**

A total of 731 potential articles were reviewed, and 132 were included based on the inclusion criteria and exclusion criteria. Some steps of the knee arthroplasty procedure were assisted and improved by using AI-based tools. Before surgery, machine learning was used to aid surgeons in optimizing decision-making. During surgery, the robotic-assisted systems improved the accuracy of knee alignment, implant positioning, and ligamentous balance. After surgery, remote patient monitoring platforms helped to capture patients’ functional data.

**Conclusion:**

In knee arthroplasty, the AI-based tools improve the decision-making process, surgical planning, accuracy, and repeatability of surgical procedures.

## Introduction

Artificial intelligence (AI) refers to machine algorithms giving the ability to reason and perform cognitive functions [1]. Over the past 70 years, AI has evolved rapidly, with computer models and algorithms designed to replicate human intelligence and performs specific tasks within various industries [2, 3]. Surgeons are key stakeholders in adopting AI-based technologies for medical care. Health-care professionals can help data scientists and engineers develop clinically relevant software.

In orthopedic surgery, the AI technology enables surgeons to provide patient-specific knee arthroplasty in clinical decision making, preoperative health optimization, resource allocation, decision support, and early intervention. However, the safety and effectiveness of AI-based knee arthroplasty are still challenging. A rigorous validation process and a clinical relevance analysis are required with new technologies. This process aims to distinguish which AI-based tool is clinically relevant and which is just hype. Many studies reporting the interest of AI-based tools in the orthopedic field have been published during the past years with the growing interest in AI-based tools in knee arthroplasty. Nevertheless, the interest and the understanding for AI in knee arthroplasty remain little-known and underused.

This study aimed to review the published articles on the comprehensive clinical relevance of AI-based tools used before, during, and after knee arthroplasty.

## Material and methods

### Article identification and selection process

In May 2021, we performed a query to identify available articles describing AI tools for knee arthroplasty pre-, intra-, and postoperatively. We searched PubMed, EMBASE, and MEDLINE databases from 2000 to 2021 using the 2009 Preferred Reporting Items for Systematic Reviews and Meta-Analyses Protocol (PRISMA). We used the following terms: “knee arthroplasty” or “knee replacement”; “artificial intelligence” or “predictive model” or “predictive modeling” or “analytic model” or “machine learning” or “remote patient monitoring” or “augmented reality” or “mixed reality” or “virtual reality” or “robotic” or “robotically-assisted”.

The inclusion criteria were English language studies reporting on AI tools in knee arthroplasty. The exclusion criteria were: (1) editorial articles; (2) systematic reviews or meta-analyses; and (3) studies evaluating joints other than the knee. Two investigators independently reviewed the abstracts of the identified articles. Discrepancies were settled by discussion between the reviewers or consultation with a third reviewer. Articles were excluded if the title and abstract did not include AI tools used in knee arthroplasty. Full-text articles were assessed if necessary.

### Definition and description of AI tools

The tools evaluated in this analysis include different sub-groups of AI tools defined and described below: Predictive modeling is a discipline of AI where algorithms generate estimates for a defined target output. Predictive models are “trained” to identify relationships between a set of features (*e*.*g*., age, body mass index (BMI), sex) and the target (*e*.*g*., the occurrence of myocardial infarction) [4]. Statistical models (*e*.*g*., regression models) and machine learning techniques (*e*.*g*., random forest models or neural networks) are used to learn the target-predictors relationship among the data [5]. In its most simplistic form, predictive modeling involved using real-world data sets to predict or estimate an outcome. Machine learning allowed a computer to utilize partial labeling of data (supervised learning) or the structure detected in the data itself (unsupervised learning) to explain or make predictions about the data without explicit programming. Deep learning models (*e*.*g*., neural networks with several hidden layers) have seen wide success in image recognition and classification where the input is represented by unstructured data (*e*.*g*., pixel values) [6]. Predictive models were usually deployed in contexts where the measurement of the output is complicated, time-demanding, expensive, or when an early estimate of the target can trigger a proactive intervention to modify the course of action and, for instance, avoid adverse events (*e*.*g*., a readmission to the hospital). The predictive models and machine learning can be used in several domains in surgical management (decision-making, aid to surgical planning).

Natural language processing aimed to understand human language and was crucial for large-scale content analyses such as electronic medical record data such as physicians’ narrative documentation [*e*.*g*., data collection of clinical scores after TKA]. Computer vision described machine understanding of images and videos, and advances have resulted in machines achieving human-level capabilities in object and scene recognition [*e*.*g*., screening of implants loosening on radiographs]. More recently, digital technologies used in augmented and mixed realities have been developed to interact with the human senses. These technologies enabled user projection into a reality described through a digital memory. Augmented reality (AR) technologies aimed to introduce virtual elements into the user’s environment [*e*.*g*., superimposition of the values of bone resection axis and the virtual bone cuts onto real-knee surfaces during total knee arthroplasty (TKA)] by measuring and understanding the user’s reality, processing, and then computing the information required, and finally rendering it to project this information to the user in correlation with reality. Mixed reality presents the surgeon with holographic elements that align with the real world, and the surgeon can manipulate the digital content generated by the mixed reality device.

## Results

**Fig. 1:**
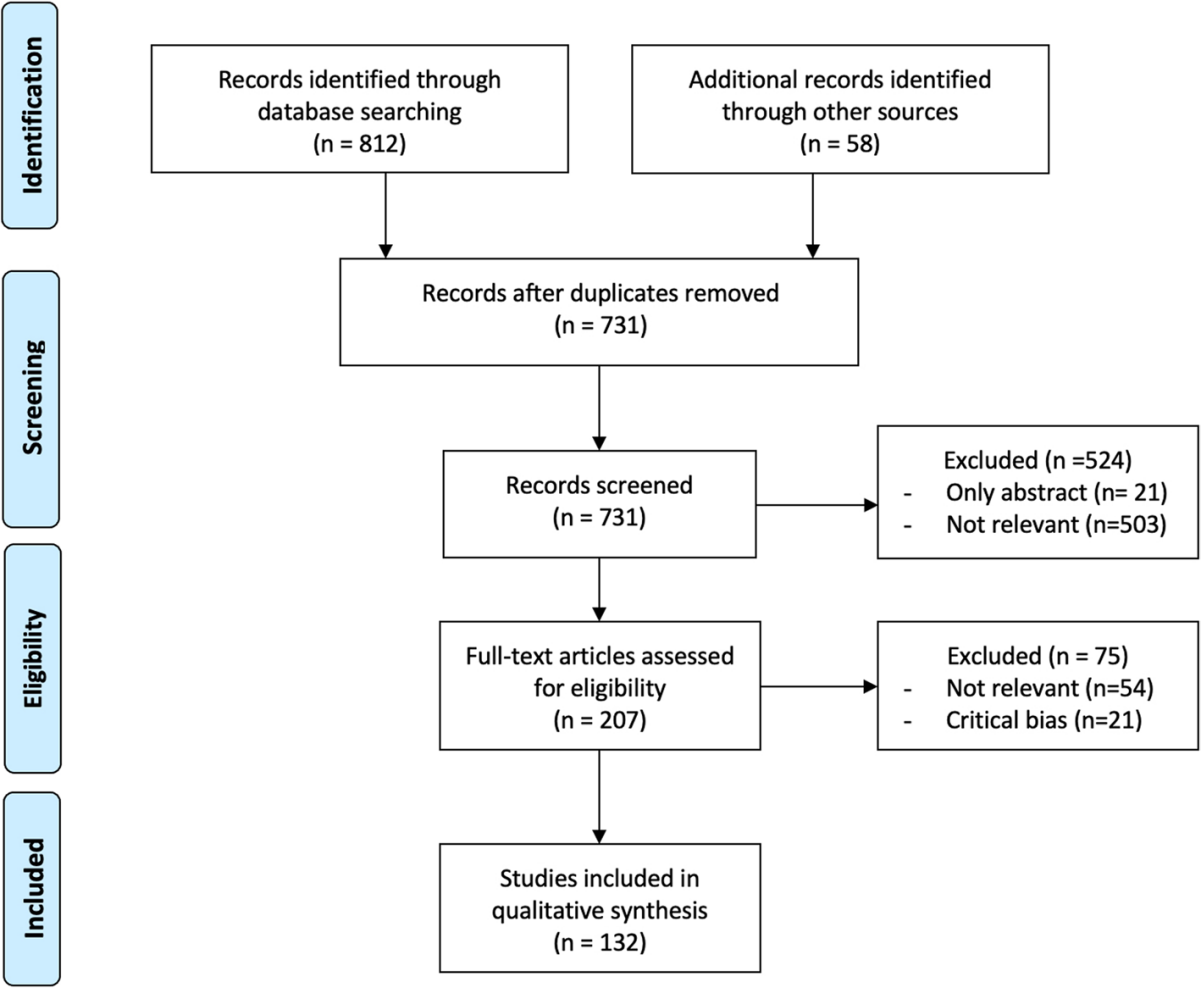
Flow chart showing initial literature search for data extraction from the final list of included studies

The PRISMA flow diagram shows the study selection (Fig. 1). Of 731 abstracts, 21 were excluded due to the lack of a full-text article, and 503 irrelevant abstracts were also excluded. Of 207 full-text articles, 54 irrelevant articles were excluded, and 21 articles with a serious risk of bias were also excluded [7]. Finally, 132 articles were included in this study.

## Review and discussion

Based on the 132 articles, we reviewed AI used before, during, and after knee arthroplasty.

### AI used before knee arthroplasty

**Fig. 2:**
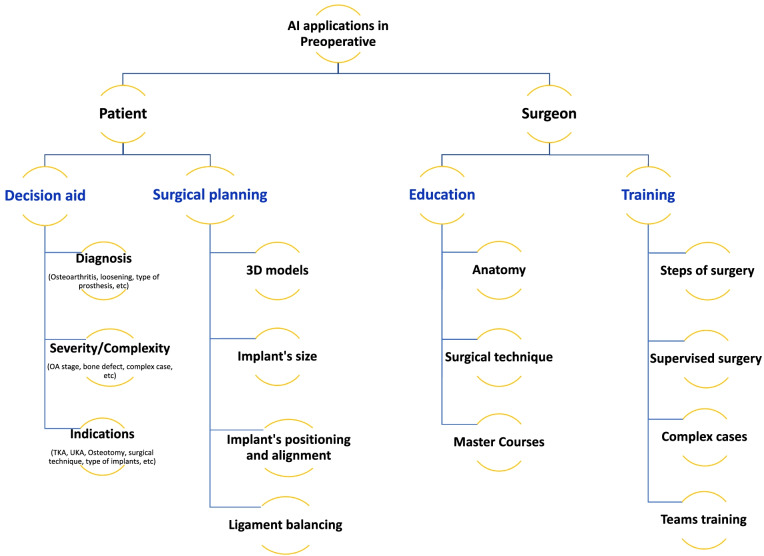
Structure chart resuming the preoperative major AI applications

Patient selection is the first priority for successful knee arthroplasty. However, 33% of patients may be improperly selected, resulting in unsatisfactory outcomes [8]. The American Academy of Orthopedic Surgeons develops the best practice guidelines and machine learning-based algorithm to standardize the patient selection process based on the medical history, symptom severity, osteoarthritis severity, failures of previous treatment, *etc* [9, 10]. The predictive algorithm models require effective communication between the patients and surgeons to develop a relationship that promotes the integration of patient preferences, values, and needs, with the transfer of knowledge regarding treatments, risks, benefits, and alternatives before making informed decisions [11, 12]. The decision-making process is based on relevant and validated predictive factors, for example, demographics or preoperative patient-reported outcome measurements (PROMs) [13]. The AI-based algorithms can be used to specify the preoperative parameters, grade severity of knee osteoarthritis, and reduce inter-observer variability [14]. In revision knee arthroplasty, it can be used to diagnose prosthetic loosening on X-ray (with a precision of > 95%) and identify implant models [15]. (Table 1 and Fig. 2).

**Table 1:** Diverse systems of AI in knee arthroplasty management

Authors	Patients	Year	Type	Time	Assessment	Factors	Conclusion
Patient Decision Aid							
Ramkumar *et al*. [16]	175,042	2019	Predictive of perioperative parameters (ANN)	Preop	Predict LOS, inpatient discharge Propose a risk-based plan for complex cases	Preop variables	Model can predict perioperative management
Bansback *et al*. [17]	280	2019	Patient Decision	Preop	Decision quality	PROMS, demographics	Predictive model with decision aid
Jayakumar *et al*. [18]	150	2020	Patient Decision	Preop	Decision quality, patient outcomes	PROMS, demographics	Presentation of RCT
Shah *et al*. [15]	697	2020	Patient decision	Preop	TKA loosening	Preop radiographs	Detection of implants loosening THA > TKA (Se 70%, Sp 96%)
Jayakumar *et al*. [19]	129	2021	Patient Decision	Preop	Decision quality, patient experience, functional outcomes	Education, preference assessment, PROMs	Better decision quality, satisfaction, improved PROMs
Yi* et al*. [14]	237–274	2019	TKA identification	Preop	Difference of TKA, UKA	Radiographs	Identification of TKA on X-ray and distinguish 2 models of TKA
Karnuta *et al*. [20]	424	2020	TKA identification	Preop	TKA models	Radiographs	Valid
Schwartz *et al*. [21]	326	2020	OA classification	Preop	OA stage	Preop radiographs	Convolutional neural network (CNN) and classify knee OA
Surgical training							
Aim *et al*. [22]	330	2016	VR training in arthroscopy	Preop	Review		Few assessments of VR training but promising
Goh* et al*. [23]		2021	VR and AR training in knee arthroplasty	Preop	Review		Few assessments of VR training but promising
Preoperative planning							
Wallace *et al*. [24]	382	2020	PM Implant Size	Preop	Component size prediction	Sex, height, weight, age, and ethnicity	More accurate than radiographic templating
Kunze *et al*. [25]	17,283	2021	PM Implant size	Preop	Component size prediction	Demographic variables (age, height, weight, BMI, sex)	Good to excellent performance for predicting TKA component Size. Main factor: sex Free app: https://orthopedics.shinyapps.io/TKASizing_Calculator/
Li *et al*. [26]	200	2021	3D reconstruction	Preop	AI-based 3D model construction	CT scan	As accurate as operator reconstruction. Faster than operator construction
Surgery							
Tsukada *et al*. [27]	10	2019	Augmented reality in surgery	Intraop	Tibial bone resection with AR	AR-KNEE system	Insufficient accuracy of bone cuts
Pokhrel *et al*. [28]	15	2019	Augmented reality in surgery	Intraop	Accuracy of bone cuts	Augmented reality system	Reliable accuracy
Verstraete *et al*. [29]	479	2020	ML PM	Intraop	Intraop planning (load)	Intraop alignment – tibiofemoral load	Validated ML algorithm
Remote patient monitoring							
Chiang *et al*. [30]	18	2017	Patient Monitoring	Postop	APDM sensors	Postop ROM	Continuous monitoring of ROM progress after TKA
Kang *et al*. [31]	60	2018	Patient Monitoring	Postop	Rehabilitation training instrument NEO-GAIT	VAS, ROM, HSS	NEO-GAIT plays more active and effective role in promoting rehabilitation after TKA
Ramkumar *et al*. [32]	25	2019	Remote Patient Monitoring	Postop	Feasibility – ROM – PROMs – exercise compliance	RPM mobile application	Pilot study — acquisition of continuous data
Mehta *et* *al*. [33]	242	2020	Remote Patient Monitoring	Postop	rate of discharge to home and clinical outcomes after hip or knee arthroplasty.	RPM mobile application	No significant difference in the rate of discharge to home. Significant reduction in rehospitalization rate with RPM
Bovonratwet *et al*. [34]	319	2020	NLP	Postop	Satisfaction	Patient narratives	Not efficient
Sagheb *et* *al*. [35]	20,000	2020	NLP	Postop	Identify data in OR report	OR report	NLP algorithms efficient

*AR* Augmented reality, *ML* Machine learning, *NLP* Natural language processing, *OA* Osteoarthritis, *OR* Operating room, *PROMs* Patient-reported outcome measurements, *PM* Predictive monitoring, *RCT* Randomized control trial, *ROM* Range of motion, *RPM* Remote patient monitoring, *SDM* Shared decision-making, *THA* Total hip arthroplasty, *TKA* Total knee arthroplasty, *UKA* Unicompartmental knee arthroplasty, *VR* Virtual reality

Surgical training is traditionally performed on real patients in the operating room and supervised by senior surgeons. It is undergoing substantial change with AI development. Immersive virtual reality is an AI-based teaching tool that provides all access levels to many surgical techniques in a 360-degree viewing mode. The surgeons can simultaneously evaluate decisions on implant choice and placement, track procedural errors and efficiency, minimize completion time, and use adjunct operating equipment (such as fluoroscope) or instruments (such as retractor). By obtaining technical and cognitive practice, the surgeons can reduce implant mal-alignment and surgical complications in primary and revision knee arthroplasty [36]. Using the assistive mode, the surgeon can obtain feedback on key steps (planning bone resection, implant positioning, sizing, assessing the virtual range of motion, and gap balancing) to anticipate difficulties or achieve a surgical target [23]. Immersive virtual reality has advantages. The surgeons can obtain learning experience without direct supervision and collect data all along the training process. The replicate real-life procedures do not carry the risk of injury or costly resources (*e*.*g*., cadavers). The downsides included limited image quality, degree of presence, cyber-sickness, haptic realism, device-related issues (*e*.*g*., battery capacity and wireless technology), and access/cost considerations [22, 37, 38].

Preoperative planning and modelization include limb alignment, implant positioning, gap balancing, and implant size. Preoperatively, proper TKA component size can be predicted by using the formulas based on the demographic data such as sex, height, weight, age, ethnicity/race, and shoe size. Still, the limited predictive factors and limited size of certain products are the drawbacks [39–41]. Demographic-based multivariate linear regression models can be used to predict more accurate implant size than digitally-templated sizes for femoral (*P* = 0.04) and tibial (*P* < 0.01) components [24]. The regression models are created using the stochastic gradient boosting model, allowing users to input data and receive individualized sizing predictions and explanations [25]. This application is made freely accessible at the following link: https://orthopedics.shinyapps.io/TKASizing_Calculator/.

Segmentation tools are developed to reduce operative time and human involvement. The tools consequently reduce the process cost and are more efficient than the usual operator [26]. The tools can be used in knee arthroplasty, spine surgery, and trauma surgery based on the three-dimensional (3D) models obtained from bone-mapping (imageless system), CT scans, and specific X-rays (image-based system) [42, 43]. As a result, more accurate component alignment, ligament balance, and implant size prediction are achieved in robotic-assisted TKA than image-less robotic-TKA [42, 44, 45].

### AI used during knee arthroplasty

**Fig. 3:**
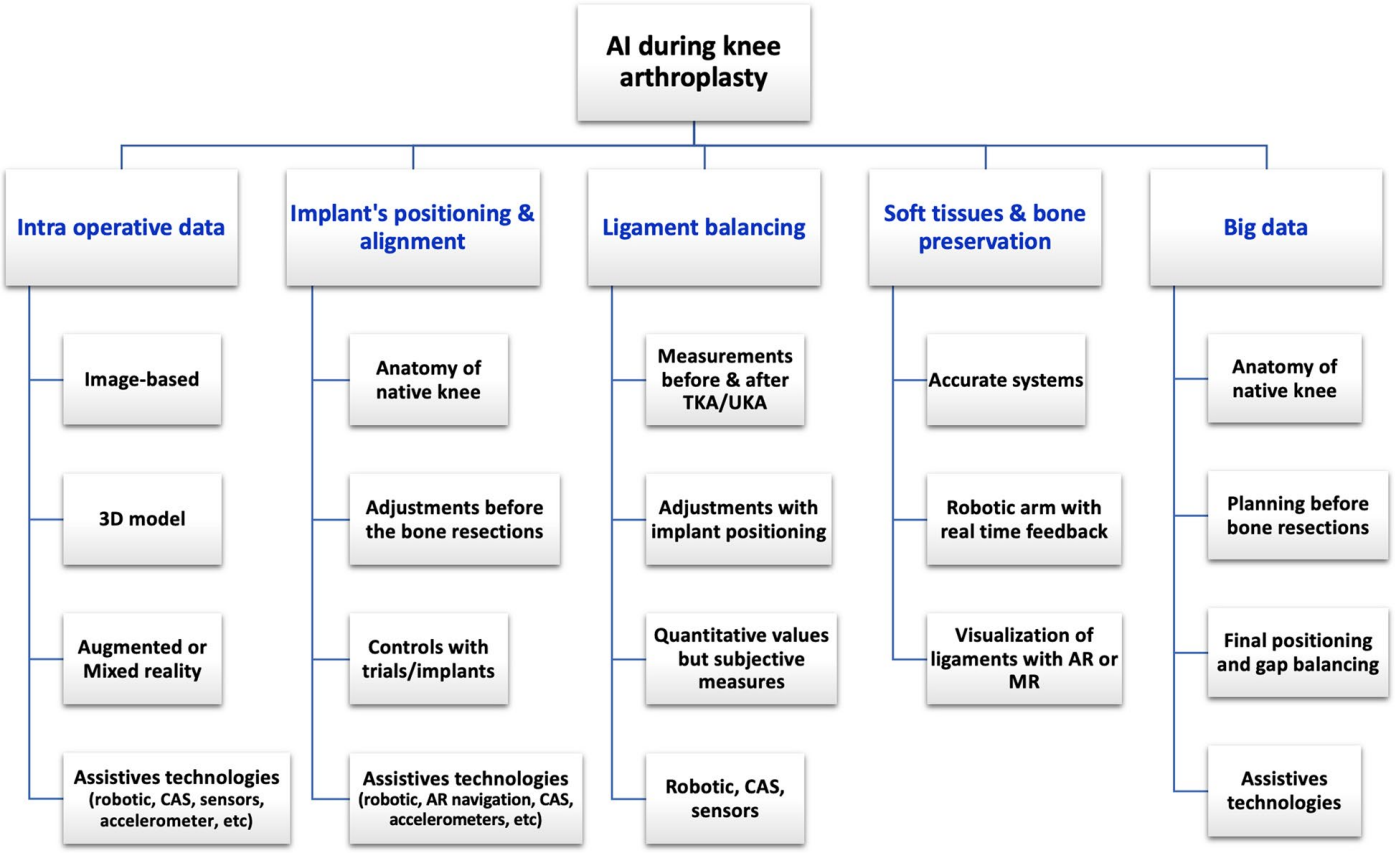
Structure chart presenting the intraoperative major interests of AI tools

Robotic-assisted knee arthroplasty is defined as machine capable of automatically carrying out complex actions, especially programmable by a computer. This system integrates information from preoperative imaging or intraoperative surface mapping, specific bone landmarks (bone shape, tibial and femoral alignment) and the ligament balancing intraoperatively. There are three categories of robotic systems, *i*.*e*., passive, semi-autonomous, and autonomous robotic systems. A passive system provides a 3D virtual model allowing accurate preoperative planning but does not prepare the bone. The autonomous and semi-autonomous systems incorporate safeguards against bone removal beyond the 3D plane. The semi-autonomous robotic-assisted system combines the benefits of a navigation system and an autonomous robotic system, and is a typical example of an AI-based tool. The collected data include bone and implant alignment algorithms and soft-tissue balance to propose surgical planning, secondarily adjusted according to the surgeon’s requests and targets. A robotic arm allows performing bone resections or positioning a cutting guide with a real-time automatic feedback system following knee movements or cut progression. Progressively, the algorithms of robotic system integrate machine learning models to improve surgical planning according to the previous surgery. During surgery, a feedback loop is created when bone cutting is controlled or the cutting guide is positioned. This control improves the surgeon’s accuracy and decreases the risk of errors. The robotic systems do not aim to replace surgeons but to be an accurate and consistent delivery tool. The major benefit of robotic systems is accurate and reproducible bone preparation thanks to the robotic interface, regardless of the system used [42, 46]. Most currently available robotic platforms assess the ligament balancing according to intraoperative bone cutting and implant positioning. The advantages of robotic-assisted TKA are accurate knee alignment, implant positioning, ligamentous balance, and soft tissue protection [47–50]. Most controlled studies suggested better short-term functional outcomes than mid- or long-term outcomes, compared to the conventional TKA [51–56]. The downsides of robotic-assisted TKA include cost-related capital investment and the consumables in the operating room, an amount of surgeon and staff’s education to optimize safety and efficiency of robotics, unpractical specific hardware with bony trackers and a bulky robotic unit, longer operative time, learning curve required, and compromised cost-efficiency. Moreover, a robotic system is usually compatible only with one type of implant. The laxity assessment at the beginning of surgery is manual and thus lacks accuracy. (Table 1 and Fig. 3).

Augmented reality-based navigation systems superimpose clinical information into the surgeon’s sight and have been developed to guide TKA implantation. Augmented reality platforms require three processes, *i*.*e*., tracking, computing, and visualization. Tracking of object position is achieved by semi-contact, or contactless methods. The semi-contact system involves attachment to the anatomy and marker and a contactless link between the features and cameras. In the contactless systems, tracking is done without the need for attachment to the patient and has been made possible with the apparition of depth cameras [57]. The computing requires to register the anatomical features tracked with the preoperative images, and to compute the clinical index from raw information, which compares the actual situation with the preoperative plan. The visualization produces an image for the user. The digital image must align with surgeons’ reality. Compared to the robotic-assisted systems, the advantages are a smaller physical footprint, a lower cost, the ability to have the intraoperative data in the same field of view, the absence of intraosseous trackers, and an easier workflow [58]. In a study examining an AR-assisted system, the preoperative CT scan was superimposed on the bone exposed during the surgery [28]. This system was found to be accurate in a cadaveric pilot study. The Pixee Medical system is a computer-assisted orthopedic surgery solution using AR to support TKA (Pixee Medical, Besancon, France). The connected glasses precisely calculate the 3D coordinates of the instruments thanks to the analysis of their specific markers (QR-Code), filmed by the integrated camera. The navigation information is displayed in the surgeon’s field of vision, which interacts with the application thanks to the glasses’ accelerometers. The NextAR™ system (Medacta, Castel San Pietro, Switzerland) requires sensors to be anchored to the femur and tibia using pins inserted within the surgical wound. A preoperative plan is generated based on CT imaging and a dedicated algorithm used to identify ligament origin and insertion to monitor balance during intraoperative navigation. To date, to our knowledge, no clinical studies have yet been published on the accuracy and the clinical efficiency of these novel devices. Despite a current important mediatization, whether these devices lead to improved patient outcomes and/or are cost-effective remains unclear.

### AI used after knee arthroplasty

Remote monitoring via smartphones can be used to obtain continuous subjective and objective data postoperatively. The first platforms have been limited by the absence of interconnectivity between applications, poor user engagement, high cost of sensors, and inability to scale [30, 31, 59]. Recently, a machine learning-based remote patient monitoring system for smartphones has been developed. These devices allow for real-time tracking of patient participation in physical therapy and home exercise programs through the patient’s smartphone. The surgical team can thus follow their rehabilitation progress and intervene with an additional clinic visit or a phone call if patients are not meeting postoperative milestones [60, 61]. A pilot study of 25 patients who underwent TKA has been validated, demonstrating the ability of this technology to passively collect data from each patient’s smartphone without interruptions [32]. A recent randomized clinical trial on 242 patients operated on hip or knee arthroplasty found no significant difference in the rate of discharge to home between the usual care arm and the remote patient monitoring arm, but a statistically significant reduction in rehospitalization rate in the remote patient monitoring arm [33].

Predictive models and machine learning can be used to estimate postoperative improvement and patient satisfaction and to learn for future patient and surgeon decision-making [62]. The increasing availability of large digital healthcare datasets facilitates the development of predictive models for postoperative outcomes after TKA (Table 2). These predictive models examine how variables such as patient-specific attributes, functional scores and preoperative pain [95], comorbidities [96], psychological features [63, 97, 98], socioeconomic indicators [63] or perioperative recovery location influence clinical outcomes. The main preoperative predictive factors are pain scores (VAS and back pain), knee specific PROMs (such as KOOS and WOMAC), range of motion, quality of life PROMs (EQ-5D), and mental health (assessed by anxiety and depression scales and SF-12). Other factors that have also been evaluated are comorbidities (ASA score), demographic data (such as BMI, sex, age), previous knee surgery, the severity of osteoarthritis, and preoperative knee alignment [99]. Predictive models using data from very large populations, including several centers or countries, and objective preoperative 3D anatomy assessment are more reliable than those built on limited data sets [78]. So far, none of the available predictive models have replicated surgeon clinical acumen [100] or become a practical tool for clinical use yet. These predictive models are still in their research/pre-clinical phase [101].

**Table 2:** Predictive models for knee arthroplasty management

Authors	Patients	Year	Type	Assessment	Factors	Conclusion/Algorithms
Judge *et al*. [63]	1991	2012	PM	Satisfaction, OKS	Age, sex, BMI, Primary diagnosis, ASA score, Index of Multiple Deprivation, OKS, EQ. 5D	Strongest determinants of outcome: pain/function (less severe preop disease obtain best outcomes); diagnosis in relation to pain outcome (RA > OA); deprivation (poorer areas = worse outcomes); anxiety/depression (=worse pain)
Lungu *et* *al*. [64]	141	2014	PM	WOMAC	5 preoperative WOMAC questions: difficulty of taking off socks, getting on/off toilet, performing light domestic duties and rising from bed as well as degree of morning stiffness after the first wakening	Predictive rule, based on 5 preop WOMAC questions
Dowsey *et al*. [65]	615	2016	PM	WOMAC (using OMERACT-OARSI responder criteria)	BMI, radiographic degree of OA (K.L. scale), WOMAC, SF-12, sex, age, ASA score, Charlson comorbidity, smoking status, etiology, SEIFA, rurality, contralateral TKA, constraint, patella, computer navigation, LOS, discharge destination, complication/adverse event	Better probability of clinical response with lower BMI, lower SF-12 MCS disability level, lower K.L., higher (worse) preoperative WOMAC
Pua *et al*. [66]	1096	2016	PM	Walking limitations (time before severe difficulty)	Age, BMI, hypertension, fall history, walking aids, contralateral knee pain, reconstruction specialist, walking ability, fast gait speed and knee pain, sex	Lower risk of walking< 15 min with younger age, lower BMI, no HTA, less fall history, less preop walking aids, no contralateral knee pain, adult reconstruction specialist surgeon, better preop walking ability, faster 1-month gait speed, lower 1-month knee
Van Onsem* et* *al*. [67]	113	2016	PM	KSS satisfaction score	Questions selections based on KOOS, OKS, PCS, EQ-5D, KSS, age and sex	Algorithm: Satisfaction at M3 = 26.10 + 2.3*sex+ 0.13*age + 1.58*Q3–1.40*Q4–1.08*Q5–0.75*Q6–1*Q7–1.12*Q8–0.88*Q9–1.10*Q10
To *et al*. [68]	737	2017	PM	Transfusion	Preop variables	Valid
Garriga* et al*. [69]	221	2018	PM	Non-satisfaction	Demographic preop pain, function	Country dependent
Shim *et* *al*. [70]	721	2018	PM	OKS (score less than 26 classified as poor).	OKS, chronic widespread pain, high expectations of knee pain after recovery, lack of active coping	Better (higher) postop OKS with better preop OKS, less chronic widespread pain, lower expectations of knee pain after recovery, better active coping strategies
Kunze *et* *al*. [71]	484	2018	PM	Satisfaction after TKA		97.5% sensitivity, 95.7% VPN
Navarro *et* *al*. [72]	141,446	2018	PM	LOS, Cost	Age, race, sex, comorbidity scores	Excellent validity
Sanchez *et* *al*. [73]	1649	2018	PM	OKS	Age, sex, marital status, Index of Multiple Deprivation, BMI, anxiety/depression, OKS, ASA score, etiology, previous knee arthroscopy, flexion contracture, ACL status	Better (higher) postoperative OKS with better (higher) preoperative OKS, no anxiety/depression (E.Q. 5D-3L Q5), fit and healthy ASA grade, no other conditions affective mobility, no previous arthroscopy, lower IMD 2004 score, lower BMI, presence of fixed flexion deformity, damaged/absent ACL, females aged < 80 or males aged > 60.
Van Onsem *et* *al*. [74]	57	2018	PM	KOOS, KSS, OKS	Preop ROM, quadriceps and hamstring force, sit-to-stand test, 6-min walk test	High postop PROMs showed higher postop functional outcomes. A model to predict the cluster allocation contained sex, ROM improvement and 6MWT improvement (sensitivity 91.1%, specificity 75%)
Calkins *et al*. [75]	145	2019	PM	Satisfaction (KSS satisfaction subscale, score less than 20 classified as unsatisfied).	KOOS, OKS, PCS, EQ-5D, new KSS, age, sex, diagnosis, previous surgery on knee, BMI, radiographic degree of OA, coronal alignment	Higher KSS score with male sex, older age, higher pain (EQ-5D-5L Q4), less knee joint stiffness (KOOS Sy1), less grinding/clicking noise (KOOS Sy4), knee felt ‘normal’ (KSS: Symptoms Q3), less awareness of knee problem (KOOS Q1), less anxiety/depression (EQ-5D-5L Q5), pain not on mind (PCS Q9), less worried about serious problem occurring (PCS Q13)
Zabawa *et al*. [76]	203	2019	PM	Patient dissatisfaction following TKA	KOOS, OKS, PCS, EQ-5D, new KSS, age, sex, diagnosis, previous surgery on knee, BMI, radiographic degree of OA, coronal alignment, payment method, education, income, diabetes mellitus, HTA, hyperlipidemia, insurance provider, comorbidities	External validation of a new prediction model; Less pain prior to surgery (Q3), lesser anxiety/depression prior to surgery (Q9) and better ability to control pain symptoms (Q9); Also found lower BMI and past medical history of hypertension through additional analysis
Twiggs *et al*. [77]	330	2019	PM	Knee pain	Age, sex, KOOS items, back pain, occurrence of hip pain, occurrence of falls in past year	Predictive model with a web application KOOS: activities of daily living, pain and symptom subscores, pain when pivoting on knee, pain when standing, difficulty bending the knee fully, frequency of back pain, severity of back pain, occurrence of hip pain, occurrence of falls in preceding year, age, sex
Tolk *et al*. [78]	7071	2019	PM	Residual symptoms (pain at rest and activity, sit-to-stand movement, stair negotiation, walking, performance of activities of daily living, kneeling and squatting)	Age, sex, ASA score, BMI, smoking, previous knee surgery, Charnley score, KOOS-PS, OKS, EuroQoL 5D-3L, NRS	Predictive model for residual symptoms
Kunze *et al*. [71]	484	2019	PM	Patient-reported health state, KSS, ROM, satisfaction = > Knee survey score	BMI, drug allergies, osteophytes, soft tissue thickness, flexion contracture, diabetes, opioid use, comorbidities, previous knee surgery, surgical indication, smoking	Knee survey score on 110 pts; 4 risks of experiencing postoperative dissatisfaction: Score 96.5–110 = low risk Score 75–96.4 = mild risk Score 60–74.9 = medium risk Score < 60 = high risk
Huber *et* *al*. [79]	34,110	2019	PM	EQ-VAS (MID), OKS (MID).	All 81 variables in NHS dataset (April 2015 – March 2016); including sociodemographic information such as living status, age groups, sex, disease affliction, EQ-5D-3L, EQ-VAS, OKS scores	Preop OKS score, often limping (OKS Q6), preop EQ-VAS, revision surgery, no disability, not interfering with work (OKS Q9), no previous knee surgery, no diabetes, extreme difficulty doing shopping (OKS Q11), age 50–59
Gronbeck *et al*. [80]	61,284	2019	PM	Inpatient admission after TKA	Demographic, comorbidity, perioperative variables	Reliable identification of candidates for inpatient admission
Bini *et al*. [5]	22	2019	PM	PROMs	35 variables (PROMS, demographic …)	Valid
Jo* et al*. [81]	1686	2019	PM	Transfusion after TKA	43 preop variables	Validated – good performance
Pua *et al*. [82]	4026	2019	PM	Walking limitation	Socio-demographic data outcomes	Better (higher) postop score with lower preop knee pain levels, lower preop depression levels, lower preop knee flexion range and Chinese race
Itou *et al*. [83]	50	2020	PM	satisfaction	KSS FJS12	Low utility
Li *et al*. [84]	1826	2020	PM	LOS	ASA, diabetes, comorbidities, anesthesia, operation time	LOS prediction model for TKA
Kunze *et* *al*. [85]	430	2020	PM	Dissatisfaction after TKA	Demographics, medical history, flexion contracture, knee flexion, outcome scores	Good discriminative capacity
Turcotte *et al*. [86]	2266	2020	PM	Ambulatory surgery for TKA	Demographics, comorbidities	Good validity
Harris *et al*. [87]	587	2020	PM	PROMs Improvement	PROMs health data	Improve decision support and decision making
Goltz *et al*. [88]	10,155	2020	PM	Risk prediction of TKA for discharge location	45 variables (sociodemographic data, postop labs, comorbidity)	Excellent accuracy to predict discharge location
Farooq *et al*. [89]	897	2020	PM	Satisfaction	15 variables (sociodemographic – surgery)	Valid - multifactorial
El Galaly *et al*. [90]	25,104	2020	PM	Revision TKA	Patient’s characteristics and surgical information	Inable to predict revision
Anis *et al*. [91]	5958–2391	2020	PM	LOS, 90 days readmission, PROMs	Age, sex, BMI, race, educational level, smoking, comorbidities, KOOS items, 12PCS, 12MCS	Scalable predictive tools Can accurately estimate the likelihood of improved pain, function, and quality of life 1 year after TKA as well as LOS and 90 day readmission.
Ko* et al*. [92]	5757	2020	PM	Acute kidney injury	18 variables	6 major variables – valid
Andersen *et al*. [93]	538	2021	PM	Revision TKA	Age, EQ-5D, comorbidities	Partially validated
Han *et al*. [94]	1298	2021	PM	LOS	36 variables	Valid

*BMI* Body mass index, *EQ-5D* Euro QOL score, *KOOS* Knee injury and osteoarthritis outcome score, KSS Knee society score, *LOS* Length of stay, *OA* Osteoarthritis, *OKS* Oxford knee score, *PCS* Pain catastrophizing scale, *PM* Predictive model, *PROMs* Patient-reported outcome measurements, *RA* Rheumatoid arthritis, *ROM* Range of motion, *TKA* Total knee arthroplasty, *WOMAC* Western Ontario and McmMaster Universities osteoarthritis index

### Limitations and future expectations

The review is not comprehensive enough to include all the available technologies but has described the basic and current AI applications in knee arthroplasty. The outputs of machine learning and AI analyses are limited by the types and accuracy of available data sets. Systematic biases in clinical data collection affect the recognition or prediction of AI patterns, such as women and racial minorities due to long-standing under-representation in a clinical trial and patient registry populations. Ethical considerations regarding the ownership and the use of AI data remain unanswered. The robotic platform storing surgeon and patient information sometimes lacks the patient’s express consent, and is then used for product development. Although the aggregate data are deidentified, who access the data and what purposes are still debatable. The European Commission has proposed a regulatory framework (released on April 2021) to monitor AI with this aim.

**Fig. 4:**
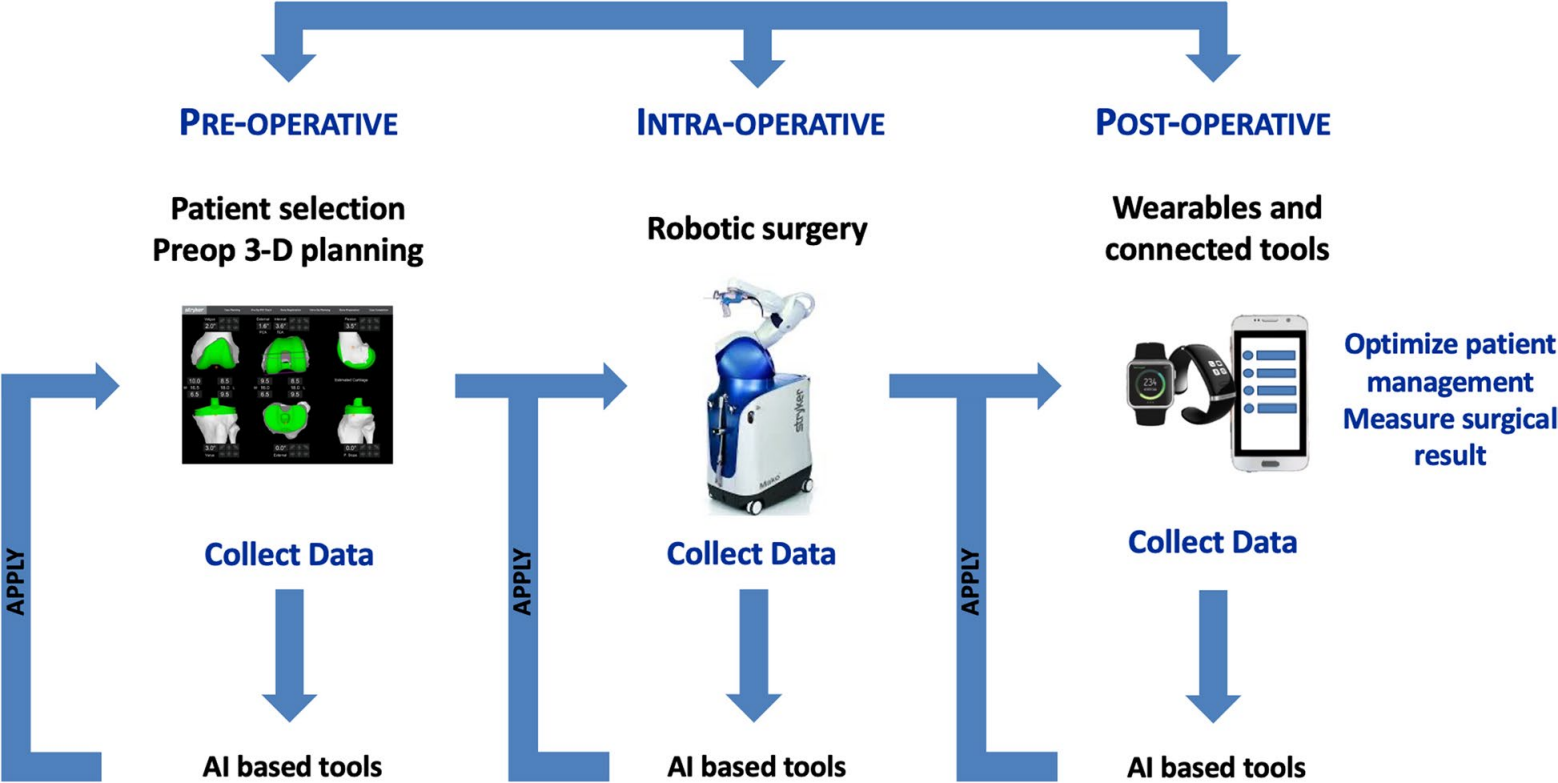
Diagram explaining the principle of the feedback loop, which interconnects data collection (before, during, and after surgery) via the connected tools to create mega data information for adjusting the surgical plan

The next challenge will be to “close the loop” using accurate interconnected data sets and predictive monitoring during the different phases of the patient path (before, during and after knee arthroplasty) to help surgeons and health-care providers in their decision-making [102] (Fig. 4). The goal is not to replace the health-care providers but to assist the medical decision collaboratively, combining the doctor’s experience and the AI-based tools. The answer is probably collaborative intelligence to adjust the patient management using predictive models and clinical experience and make the subsequent surgery better for every patient.

## Conclusion

In knee arthroplasty, the AI-based tools improve the decision-making process, surgical planning, accuracy and repeatability of surgical procedures. More clinical evidence is needed to confirm the benefits.

## Data Availability

Not applicable.

## Abbreviations

AI: Artificial Intelligence
AR: Augmented Reality
BMI: Body Mass Index
EMR: Electronic Medical Record
ML: Machine Learning
MR: Mixed Reality
NLP: Natural Language Processing
PROMs: Patient-Reported Outcome Measurements
PM: Predictive Monitoring
ROM: Range Of Motion
RPM: Remote Patient Monitoring
SDM: Shared Decision-Making
TKA: Total Knee Arthroplasty
UKA: Unicompartmental Knee Arthroplasty
